# The Modern School of Medicine

**Published:** 1896-03

**Authors:** 

**Affiliations:** Archiv. Für Homœopathie


### THE MODERN SCHOOL OF MEDICINE.

DR. VILLERS, ARCHIV. FÜR HOMŒOPATHIE, JANUARY, 1896.

[Translated by B. Fincke, M. D.]

At the beginning of the new year I tender my readers my heartiest congratulations, and to my friends I wish all that is good, but to my enemies that they may find fault with me as much as they please.

To be a homœopathic physician, a homœopathician, is not an easy matter at our day. In an external aspect we have it much easier than our predecessors. We can call in consultation every specialist. Almost everywhere we are in pleasant personal relations with the colleagues of the other school; and where a homœopathic physician finds himself isolated, a part of the fault must be laid at his own door. The necessity of uniting, which the distress of the medical profession has brought about, allows us, at least, to wipe out the internal differences toward the outside world. However, under these circumstances it is the more difficult to remain a pure homœopathician, for there is no help, notwithstanding all mutual acknowledgment, all readiness to leave personality untouched in the scientific warfare; there exists a deep gulf between the allœopathic and homœopathic school.

The traditional school of medicine, which in its way has performed enormous labors, and also lately by the acceptance of the departments of bacteriology and serum-therapy, imagines itself to have made great strides forward, is and remains for all that an antiquated school, because it has no law as basis of its actions. Therefore, also, the capacity of the individual physician depends so extraordinarily upon his talent, and upon the amount of his ability to recognize the individual traits of the pathogenetic picture. On the contrary we homœopathic physicians can denote ourselves as the modern school, though our whole method and our literature are deplorably old-fashioned.

Whoever after receiving a scholarly education in medicine approaches our labors, can in the first instance not help himself but be repelled. The statements are in such decided contradiction to what his clinical experience has taught him, and to what has been demonstrated to him *ex cathedra*, the selection of the remedy according to the symptoms appears to him so much smacking of laity that the young medical man, brimful and proud of his science, cannot take any pleasure in it. Only then, after he has gone through the school of bad experiences, when he has found out that in practice the well-sounding apodeictical assertions of his academical teachers come true to but an insignificant extent, when he has felt that the amount of help which can be afforded to the sick is very limited, and essentially does not extend beyond a support of the body in the fight against disease, only then he approaches our mode of treatment with open eyes, and even under these circumstances he will find difficulties in acknowledging the importance of our aim. At first, indeed, it will appear to him as a practical therapeutical progress which has grown up from a soil not ploughed scientifically, he will assign to Homœopathy no higher place than the hydropathy of a Priessnitz, and if at all a working spirit, he will find the need of interpreting his new knowledge and his new results by those tenets taught to him at the university, and then again he stands before an impossibility. Only when he has made up his mind to put aside entirely his whole medical knowledge, and naively and without prejudice to consider the questions which the practice and study of the pathogenetic picture offer to him, only then he arrives at the perception that he has turned to a system of medicine which represents a true actual progress, only then he feels and recognizes that he has a law as basis of his therapeutical action, viz., the law of the proportionality of the remedy to the individual pathogenetic symptoms, only then can he practically carry out what had been his ideal already in his former allopathic period—the individualization, the recognition of the individual traits in the pathogenetic picture; and now he has the power in his hands to support the body against the attacks of disease, not only as a lukewarm ally, but as a champion to combat the morbid process itself by the specific correctly selected remedy. From that moment, when this knowledge arose in his mind, he is a homœopathician; from the same moment, however, he also knows no more dealings with the old system of medicine. Only homœopathicians who have worked themselves up to this standpoint can promote our cause; all those half-way natures—those men who march, turning right and left, instead of keeping the straight line—are of no use to us; they only flatten out our doctrine and produce weaklings, as they themselves are—a feeble generation of epigones.

In this one point of knowledge of Homœopathy we are all of one mind, if even the particular questions on the most suitable form of the administration of remedies have brought about differences between us, and in this special branch (posology) we all could also work together on this great problem, which still expects its solution from us.

We have to perfect our doctrine—in which the practical knowledge far surpasses the theoretical formulation—from our own standpoint, and to create an edifice of doctrine complete in itself, in which every proportion is based upon propositions experimentally demonstrable, and with it upon a fundamental law of nature, in which we keep away from the application of foreign material.

This labor is quite colossal, for it requires a philosophical education and a knowledge of the natural science of the first rank. Before all, however, it requires the moral courage to abdicate all traditions and to acknowledge only the experiment, and the experiment again and again, as our guiding and determining method.

If we look around in the civilized world, where Homœopathy thrives and where it languishes, we find that its progress or regress depends upon the kind of its representatives. The sick public never has a predilection for any kind of system, but it prefers to be healed; and if it gains the impression that this healing is accomplished more safely and more speedily by a certain physician, it turns to him and only much later to the system to which he belongs. The man, thinking scientifically, turns to every doctrine which has been built up scientifically, and the scientific character of which can be proved by the kind of its work, but not by the conclusions which may be drawn from it.

Therefore, we need nothing for the prosperity of Homœopathy in Germany than men who are willing to work; who do not acquiesce in treating the sick merely, but who also demonstrate the results of their work—the external results as well as the internal motives—and who stand for their cause with the fascinating fidelity of conviction of an apostle. In fulfilling this duty we also fulfill our part of the duty as citizens, and of the general great philanthropic duty, to devote our best efforts of our fellow-men's welfare.

